# Role of soluble biomarkers in treating multiple sclerosis and neuroinflammatory conditions

**DOI:** 10.1016/j.neurot.2025.e00588

**Published:** 2025-04-19

**Authors:** Gauruv Bose, Simon D.X. Thebault, Giulia Fadda, John A. Brooks, Mark S. Freedman

**Affiliations:** a Department of Medicine, The University of Ottawa and Ottawa Hospital Research Institute, Canada; b Department of Neurology and Neurosurgery, Montreal Neurological Institute and McGill University Health Centre, Canada

**Keywords:** Multiple sclerosis, Biomarkers, Neuroinflammation, Neurofilament, Prediction, Diagnosis

## Abstract

Multiple sclerosis (MS) is a complex, chronic immune-mediated disease characterized by acute and progressive inflammatory damage of the central nervous system. MS manifests clinically with unpredictable neurological symptoms from focal inflammatory attacks as well as gradual neurodegeneration which contribute significantly to long-term disability progression. As treatment options advance, developing more personalized strategies capture heterogeneous mechanisms of injury which may be targeted or predict outcomes has been a focus of ongoing investigation. The role of soluble biomarkers has emerged as a pivotal tool to assist in these goals. Early promising candidates include neurofilament light chain (NfL) and glial fibrillary acidic protein (GFAP); these intermediate filaments that are expressed in neurons and astrocytes, respectively, are reliably measurable from blood samples and can reveal clinical and subclinical changes, as well as predict progression. Changes in these biomarkers can indicate a response to therapy, thus potentially be used as endpoints in clinical trials. Furthermore, recent research has identified a potential role of these and other soluble biomarkers in other neuroimmunological conditions including neuromyelitis spectrum disorder (NMOSD) and myelin oligodendrocyte glycoprotein associated disease (MOGAD), autoimmune encephalitis, neurosarcoidosis, neuropsychiatric involvement in connective tissue disorders and vasculitides, and a host of neurodegenerative conditions. By integrating biomarker analysis into routine clinical assessments, healthcare providers may move toward a more nuanced and individualized care model, better equipped to meet the challenges posed by these multifaceted diseases. Understanding the dynamics of these biomarkers has many applications that can improve personalized medicine in MS.

## Introduction

Multiple sclerosis (MS) is an incompletely understood chronic immune-mediated disorder affecting the central nervous system (CNS). Persons with MS (pwMS) are affected by inflammatory attacks which lead to demyelination, axonal damage, and gliosis that are identified by new or enlarging T2 hyperintense lesions or gadolinium contrast enhancing lesions (CELs) on magnetic resonance imaging (MRI) and can result in neurological symptoms. Clinical attacks may be followed by some degree of recovery, but they often leave pwMS with some degree of neurological deficit. In addition, pwMS may be affected by more gradual neurological injury, without discrete clinical attacks, and with or without clear activity on MRI. This form of neurological injury is thought to be caused by a combination of ongoing but subclinical attacks of inflammatory activity, smoldering lesional inflammatory activity, changes in normal appearing brain including microglial activation and other neurodegenerative processes that culminate in brain or spinal cord atrophy, and ultimately progressive neurological worsening [1]. Through these processes, pwMS may have a wide array of unpredictable neurological symptoms that eventually leads to progression. Indeed, MS is one of the most common causes of non-traumatic disability, affecting nearly 3 million people world-wide [2]. Although the treatment landscape for MS and other neuroinflammatory conditions has, and continues to, quickly expand, the use of higher intensity disease-modifying treatments (DMT) may lead to better disease control but is offset by a greater risk of immune suppression. The risk-stratification of pwMS would allow for a more personalized approach to selection of treatment [3,4] and soluble biomarkers play a key role [5].

A biomarker is any measurable indicator that reflects a disease process and can ideally be detectable before an eventual clinical outcome occurs. Soluble biomarkers are molecules that can be detected in body fluids such as cerebrospinal fluid (CSF) or the plasma or serum of blood samples. With advances in measurement techniques, very low concentrations of these proteins, in the picogram per millilitre quantities, can be reliably measured using modern assays such as single molecule array (SIMOA) or high-sensitivity robust prototype assays (Roche Elecsys®) [6,7]. In MS and other neuroinflammatory conditions these are incredibly valuable, since they offer critical insights into pathophysiology and allow the possibility of detecting, and potentially acting upon, subclinical changes that may prevent greater amounts of neurological damage and reduce ensuing disability. Among these, serum neurofilament light chain (sNfL) and serum glial fibrillary acidic protein (sGFAP) have gained prominence for their potential to reflect disease activity, progression, and treatment response. These proteins are intermediate filaments expressed in different cell types: sNfL is expressed in neurons and higher levels reflect neuroaxonal damage, while sGFAP is expressed primarily by astrocytes within the CNS and higher levels reflect gliosis and astrocyte turnover. These, and other biomarkers, not only assist in the diagnosis of MS but also provide prognostic information that can help tailor personalized treatment approaches, marking a significant shift toward precision medicine in neuroimmunology.

The role of soluble biomarkers extends beyond MS, with evidence increasingly supporting their utility in related disorders such as Neuromyelitis Optica Spectrum Disorder (NMOSD) and Myelin Oligodendrocyte Glycoprotein-Associated Disease (MOGAD). In these conditions, sNfL and sGFAP have shown promise as indicators of disease severity and neuronal damage, contributing to a more nuanced understanding of these complex diseases. Other soluble biomarkers have been investigated for their prognostic role, such as anti-MOG antibody titres. Additionally, the exploration of biomarkers in other neuroinflammatory conditions—such as autoimmune encephalitis, neurosarcoidosis, neuropsychiatric involvement of connective tissue disorders such as systemic lupus erythematosus and Sjogren's syndrome, and vasculitides such as amyloid-beta related angiitis—highlights their potential as diagnostic and prognostic tools across a spectrum of neurological disorders. By analyzing the levels of these biomarkers, clinicians can better monitor disease activity and therapeutic responses, enhancing the ability to manage these multifaceted conditions effectively.

This review paper will delve into the evidence supporting the use of soluble biomarkers in MS and other neuroinflammatory diseases, emphasizing their diagnostic and prognostic value. We will explore the current state of research on sNfL and sGFAP, along with other emerging biomarkers, to assess their impact on clinical practice and patient management. Additionally, we will discuss the future of soluble biomarkers in neuroimmunology, including potential avenues for research, economic impacts, and the integration of biomarker analysis into routine clinical assessments. As we continue to unravel the complexities of these disorders, soluble biomarkers are innovative and cost-effective tools that may transform our approach to diagnosis, treatment, and ultimately, patient outcomes in the realm of neuroinflammatory diseases.

## Role of soluble biomarkers in multiple sclerosis

**Fig. 1:**
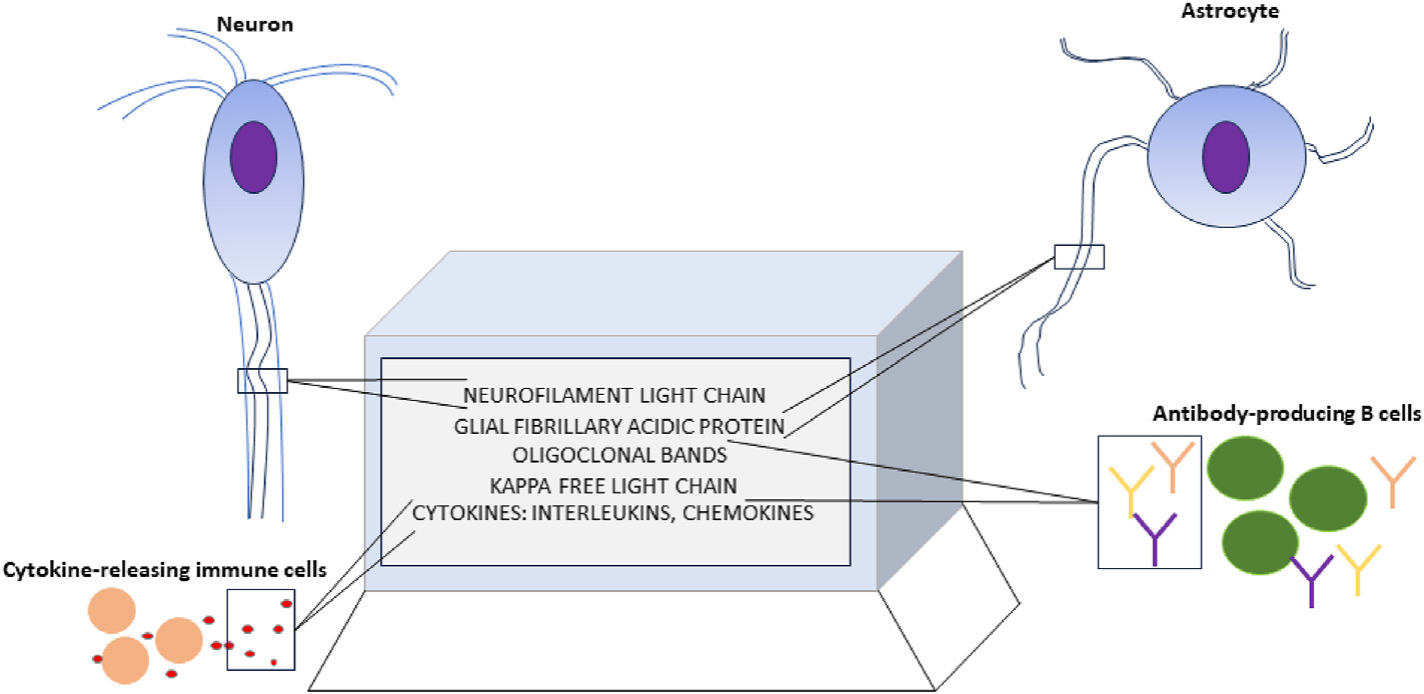
Schematic of soluble biomarkers in multiple sclerosis.

Multiple sclerosis is a relatively common immune-mediated disorder of the central nervous system that has a wide array of treatment options despite an incompletely understood pathophysiology. In addition to varied clinical features and neuroimaging findings, a variety of soluble biomarkers have had a key role in improving the diagnosis, prognosis, monitoring, and evaluating treatment response in MS (Fig. 1).

### Diagnosis of MS

The core pathophysiology of MS is incompletely understood, and although the clinical course can be quite varied between patients, it has been recognized and investigated thoroughly. The diagnostic criteria for MS, after ruling out mimicking diseases, are based on somewhat unique clinical and radiological features with patients exhibiting disease that affects multiple areas of the CNS over time (i.e. dissemination in space and time); however, the implementation of biomarkers into this framework has started [8]. Incremental improvements of the diagnostic criteria (referred to as the ‘McDonald Criteria’) have allowed for earlier identification of patients who may benefit from disease modifying treatment and has resulted in lower levels of disability in people with MS [9].

The current diagnostic criteria are based on balancing sensitivity with specificity, such that people who may have MS get the further testing and close follow-up they deserve, and people who may have other conditions receive the scrutiny and proper diagnosis and treatment they need. However, the root etiologic cause of MS is still uncertain though increasingly being investigated. A large cohort from the United States identified that infection with Epstein-Barr virus (EBV) increased the risk of developing MS by 30-fold, and that it was essentially necessary in order for MS to occur [10]. Also of interest, in the patients who seroconverted to EBV, the ones who later went on to develop MS had higher sNfL levels that those who did not subsequently develop MS, while prior to EBV seroconversion both groups had similar sNfL levels, suggesting that neuroaxonal damage only begins to occur after EBV infection took place. This cohort was analyzed further, and it was found that sNfL levels can begin to increase as early as 6 years prior to first clinical MS symptoms [11]. Furthermore, emerging data hints at a possible role for EBV-infected B cells in driving MS neuroinflammation through several plausible mechanisms, including a direct role of CNS penetrant EBV infected B cells, dysregulated T and NK responses, and molecular mimicry for example between EBV nuclear antigen 1 (EBNA1) and the glial cell adhesion molecule (GlialCAM) [12,13]. This suggests that even during a “prodromal period” before typical symptoms of MS arise, soluble biomarkers may be useful in identifying patients who have the MS pathophysiology already underway, and thus may benefit from treatment.

The McDonald 2017 revisions re-introduced a well-known soluble biomarker to be included in the diagnostic criteria for MS: oligoclonal banding (OCB). This test involves identifying two or more bands on gel electrophoresis present in CSF, but not in serum, and is related to the process of intrathecal IgG synthesis which has been reported since 1942 [14]. The clinical relevance of CSF-specific OCB presence was initially highlighted in patients with neuroinfectious disease such as neurosyphilis or subacute sclerosing leucoencephalitis, although it was also identified in patients with autoimmune neurological disorders such as neurosarcoidosis, autoimmune encephalitis, and MS [15]. Due to the non-specific diagnostic identification of OCBs, it was not included in the earlier iterations of the McDonald criteria, despite large registry analyses showing approximately 94 ​% of patients meeting the MS diagnostic criteria having CSF-specific OCB presence [16]. Including OCBs in the 2017 criteria allowed clinicians to make a formal diagnosis of MS and begin treatment without needing to wait for a patient show dissemination in time by developing a new lesion on repeat MRI, nor enduring a separate clinical attack that may leave permanent disability.

The identification of OCBs in the CSF, but not the serum, is not without its limitations. Aside from other non-MS diseases also potentially having OCB presence, it is a resource intensive task that requires a technologist to individually interpret the samples. An alternative approach is identification of intrathecal kappa-Free Light chains (kFLC), which utilizes an assay that can be automated, quick, and relatively inexpensive [17]. kFLC measurement is an alternative to OCB in the latest iteration of the McDonald criteria as a cost-effective surrogate for CSF-specific OCB presence [18].

The diagnosis of MS typically starts with a clinical syndrome that is different between individuals, though follows a particular pattern [19]. As MRI technology improved and became more widespread, the introduction of this imaging biomarker into the diagnostic criteria has become routine, despite the test in and of itself not being entirely specific. The newest 2024 update of the McDonald criteria allow for a diagnosis of MS based solely on the MRI with or without support from other biomarkers [20]. In fact, in patient with what is at present a radiologically isolated syndrome (RIS), who have the MRI changes due to immune-mediated relapsing attacks on the central nervous system, incontrovertibly are at risk for a subsequent attack that may impact more eloquent areas resulting in clinical manifestations and would benefit from treatment. A cross-sectional analysis of people with RIS from a large referral centre in Toronto, Canada, identified that elevated plasma GFAP correlated with worse imaging features such as T1-black holes and paramagnetic rim lesions, suggesting that there is underlying astrocyte turnover even in patients with seemingly no clinical features of MS [21]. Other studies have found that patients with RIS and serum NFL levels higher than 5 ​pg/mL are more likely to convert to MS, further substantiating that biomarkers may be useful even in patient without clinical symptoms but an MRI that is compatible with MS. With improved treatment for MS, the goal is to diagnose and introduce effective treatment for patients earlier, with biomarkers, in the appropriate clinical context, facilitating this process.

### Prognostication in early MS

The course of MS is highly variable, with some individuals destined for a somewhat “benign” or mild course, wherein many decades pass after first onset without acquiring significant disability; while other patients have a more “aggressive” course, and can develop significant disability, often requiring a gait aid, early after diagnosis [22,23]. There are also pwMS who develop progressive neurological disability without overt inflammatory relapses early after diagnosis; these patients may respond best to treatments that target progressive and non-relapsing mechanisms of injury in MS, for example with small molecules that cross the blood brain barrier better to target innate immune cell involvement in CNS-compartmentalized inflammation. Risk stratifying patients and predicting disease course may lead to more patient-targeted treatment plans with the most appropriate medication selected for a given patient. Once the diagnosis of MS has been made, biomarkers can also be useful in this prognostication.

Predictive models have looked at different risk factors that portend a better or worse prognosis, involving clinical, radiological, as well as biomarker data. The earliest risk stratification tools have used clinical and MRI activity to predict outcomes and identified that patients with higher disease activity earlier despite treatment are more likely to develop progressive disease [[24], [25], [26]]. Other analyses have identified that patients who develop motor deficits and experience first symptoms of MS at a later age are more likely to develop worse disability within a decade of diagnosis [27,28], while other studies have identified that those with first symptoms at a younger age are more likely to experience worse outcomes by age 50, suggesting a longer disease duration leads to great accumulation of disability in MS [29]. In one study out of the Brigham and Women's Hospital Longitudinal MS Cohort (CLIMB), 37 predictor variables were measured in 704 patients with MS to analyze their 10-year follow-up data [26]. Multiple forms of variable-selection found that using the Least Absolute Shrinkage and Selection Operator (LASSO) method, 10 LASSO-selected clinical predictor variables created a model that performed best [28]. When this model was re-run to include serum biomarkers, the authors found that adding baseline sNfL and sGFAP improved the model's area under the curve (AUC) for predicting secondary progressive MS (SPMS) at year-10 from 0.73 to 0.77 (P ​= ​0.021) [30]. Furthermore, findings supported other studies where, by 10-year follow-up, patients with higher baseline levels of sNfL [[30], [31], [32], [33]]were more likely to have higher T2 lesion volume and brain atrophy [[31], [32], [33], [34]], while those with higher sGFAP, were more likely to develop progressive disease [[35], [36], [37], [38]]. Therefore, sNfL more reliably predicates the degree of overt, temporaneous, neuroaxonal damage, while sGFAP correlates with lesion burden, brain atrophy, and progressive disease [[39], [40], [41], [42]]. The predictive importance of these biomarkers is best realized when they are measured at baseline [30,43], and may be used to identify patients who may be undergoing a more overt inflammatory, versus more covert progressive, phase of their disease.

### Active MS

A key possible role for a soluble biomarker is for monitoring patients to detect early signs of disease activity. At present, follow-up for pwMS demands regular neurological evaluations along with periodic MRI scanning [44]. However, in certain circumstances obtaining MRI may be limited and if a patient might be having symptoms, the timeliness of obtaining one might be challenging. A time-sensitive blood test may be useful in identifying patients with subclinical active disease and can rule in inflammatory activity resulting in neurological symptoms and differentiate from other causes of neurological worsening such as pseudo-exacerbation during stress or infections, migraine, or functional neurological symptoms [[45], [46], [47], [48]]. In patients with acute inflammatory attacks, sNFL has been shown to increase approximately 3 months before an attack [32,49]. Higher sNfL suggests more focal inflammatory and relapse-associated biology, anticipating the number and volume of new T1-weighted CELs and new T2-weighted lesions on MRI scans. In a cross-sectional study, pwMS with higher sNfL levels in terms of both absolute values, as well as relative to their baseline values obtained during remission, were more likely to have a CEL [50]. Regardless of subsequent treatments, higher sNfL early in the course of MS suggests of more aggressive course of disease, and higher likelihood of reaching various disease milestones such as confirmed disability worsening, reaching EDSS 4 [51]. Dynamic changes in sNfL (in addition to one-off baseline levels) is an independent predictor of impending relapse activity, where doubling in levels over time is associated with near doubling in subsequent relapse risk [52]. Serial monitoring of patients with sNfL may therefore be useful in identifying those at risk for an impending inflammatory attack, affording an opportunity for treatment optimization. The limitation at present is that sNfL testing, a quick and cost-effective test, has limited access and the turnaround time needed for intervention may not yet be widely available [5,6,53].

### Treatment response in MS

A crucial potential use of soluble biomarkers in MS is to evaluate treatment response, and by the same token biomarkers may be able to help guide treatment discontinuation. Clinical trials have shown that effective medication leads to a reduction in sNfL([40,54,55]). The corollary may well be that failure to suppress sNfL could be an indicator of ongoing tissue injury and non-relapsing progressive injury, thus an indicator of sub-optimal treatment response, thus an indicator of sub-optimal treatment response, thus an indicator of sub-optimal treatment response [56].

Rises in sNfL may not necessarily reflect ongoing inflammatory disease, as we have observed transient increases following the exposure to potentially neurotoxic chemotherapy, such as busulfan, as part of a cocktail used for immune ablation in autologous hematopoietic stem cell transplantation (AHSCT). AHSCT effectively halts all inflammatory activity from MS, yet we have seen a transient increase in sNfL levels as well as brain atrophy in the first 6 months post after transplant, which then returns to levels seen in patients without MS([57,58]).

Treatment discontinuation is a major interest in pwMS who are aging with little ongoing disease activity. Other than cladribine, alemtuzumab, and AHSCT, there have not been studies that clearly demonstrate a durable response to treatment after an initial induction followed by period off therapy. The recent phase 4, randomised, non-inferiority clinical trial investigating discontinuing disease-modifying therapies (DISCOMS) illustrated that in pwMS age over 55 who have been clinically relapse free for 5 years and radiologically activity free for 3 years have approximately a 12.2 ​% risk of new clinical or MRI activity in the 2 years following discontinuation, which did not support non-inferiority [59]. However, in this study patients were mostly on earlier treatment options as opposed to modern higher efficacy monoclonal antibodies, and soluble biomarkers were not investigated to risk-stratify patients. A different study retrospectively investigated patients who stopped their disease modifying treatment after at least 2 years of clinical and MRI stability, and had 2 blood samples drawn with a year before and after stopping treatment, and found that sNfL and sGFAP prior to stopping therapy, when patients were stable, was not indicative of subsequent disease activity; however, patients who demonstrated a greater increase in their sNfL or sGFAP after treatment-stop were more likely to exhibit clinical and MRI worsening [60]. This laboratory finding preceded patients experiencing the clinical or MRI activity, thus possibly allowing an opportunity for intervention by resuming therapy if biomarker levels begin to rise after discontinuation.

The use of soluble biomarkers in the diagnosis, risk-stratification, and monitoring of pwMS may be a more cost-effective way to identify patients who benefit from earlier treatment and potentially help with the selection of the most appropriate treatment for a specific patient.

## Neuromyelitis optica spectrum disorder and myelin oligodendrocyte glycoprotein-associated disease

### Central nervous system-directed antibodies in demyelinating conditions

Other so-called “demyelinating” inflammatory diseases of the CNS include immune-mediated disorders targeting glial cells: NMOSD and MOGAD. These conditions have substantial differences from MS in terms of pathophysiology, disease progression, and treatment response. The discovery of specific serum biomarkers to establish their diagnosis marked a transformative development in the field of demyelinating disorders, and a major shift in diagnostic approaches.

Over 80 ​% of cases of NMOSD are associated with circulating antibodies to aquaporin-4 (AQP4+ NMOSD), a water channel primarily expressed in the perivascular astrocytic end-feet. In NMOSD, the targeting of AQP4 leads to primary disruption of astrocytes in a complement-dependent manner, with secondary demyelination and neuronal damage. While AQP4 antibodies are crucial for the diagnosis of AQP4+NMOSD, the utility of monitoring of their concentration to predict impeding relapses and response to treatment is controversial, and likely limited [[61], [62], [63], [64], [65], [66]].

In MOGAD, autoantibodies targeting myelin oligodendrocyte glycoprotein (MOG), a myelin surface protein exclusively expressed in the CNS, are detected in the serum and, in a smaller subset of patients, in the CSF. Unlike the well-established pathogenic role of anti-AQP4 antibodies in NMOSD, definitive proof that anti-MOG antibodies induce demyelination in MOGAD is yet to be established, and autoreactive T cells may play a significant role in the disease's pathobiology [67]. The sensitivity and specificity of MOG antibody testing for diagnosing MOGAD are lower than for AQP4+NMOSD, and the recognition of typical clinical and imaging features remains essential to support the diagnosis when antibody titers do not reach a discriminatory threshold [[68], [69], [70]]. However, unlike AQP4 antibodies, the persistence of MOG antibodies following an acute attack has greater prognostic value, as conversion to seronegative status is associated with a reduced risk of future relapses [[71], [72], [73], [74]], and monitoring serum MOG antibody levels after diagnosis is part of clinical practice in many centers.

### Utility of sNfL and sGFAP for MOGAD and AQP4+NMOSD

The growing recognition of sNfL and sGFAP as useful biomarkers in MS has generated great interest in its potential applications for patients with MOGAD and NMOSD.

Levels of sNfL are elevated in MOGAD and NMOSD patients relative to healthy controls [[75], [76], [77], [78], [79], [80], [81], [82]]. In MOGAD, greater elevations are observed in cases with brain involvement, encephalopathy, or seizures [77,83,84]. This aligns with the extensive brain MRI abnormalities typically observed in MOGAD patients presenting with acute disseminated encephalomyelitis (ADEM) or other brain phenotypes, while presentations with isolated optic neuritis (ON) are often associated with normal brain MRI and generally weaker sNfL elevation [83]. In both, AQP4+NMOSD and MOGAD, sNfL are increased during relapses compared to remission, with levels correlating attack severity [77,78,81,[85], [86], [87]].

However, sNfL may lack specificity in differentiating MOGAD, NMOSD, and MS, particularly when measured in isolation or near an acute event [77,82]. Conversely, sGFAP concentration rises in response to astrocytic damage, and the levels measured near an acute attack are generally higher in NMOSD than in MOGAD and MS, suggesting utility for the differential diagnosis, further enhanced by the assessment of sGFAP/sNfL ratio [77,85,88,89]. However, since sGFAP levels decrease rapidly in the days following an acute attack, their diagnostic utility might be affected by the timing of sample acquisition and be greater in proximity to clinical attacks [77]. The increase in GFAP values in AQP4+NMOSD is also typically proportional to attack severity [85,88,89].

In MS, an important potential use of sNfL is as a marker of subclinical disease activity, particularly when used in serial longitudinal assessments. However, sNfL is not likely to play a useful role to this purpose in MOGAD and AQP4+NMOSD, where silent accumulation of CNS lesions and of progression of disability independent of relapses are rare [86,90]. Given the significant increase in sNfL and sGFAP concentration in conjunction with a clinical NMOSD attack, these molecules have been proposed as useful biomarkers to distinguish between true and pseudo-relapses [77,85,86,91].

At present, evidence for utility of sNfL as a predictor of relapse risk in MOGAD and AQP4+NMOSD is limited. In MOGAD, studies suggest that higher sNfL levels at onset do not correlate with an increased risk of relapse [76,80]. However, larger studies incorporating standardized sampling times, longitudinal sNfL monitoring during remission, and an assessment of the sNfL/MOG antibody ratio may provide more insights into its predictive potential [76]. In AQP4+NMOSD, most studies indicate that sNfL does not reliably predict relapses [77,85,92,93], while some studies have shown higher baseline levels of sGFAP to be associated with shorter intervals to the next attack [88,93].

Both sNfL and sGFAP decrease following immunotherapy in patients with AQP4+NMOSD([88,94,95]), while the effect of treatment on NfL levels in MOGAD is less clear [77,83]. Further studies are needed to validate the utility of these biomarkers in monitoring treatment response in both conditions.

### Additional biomarkers in MOGAD and NMOSD

As fluid biomarker research advances, new candidate molecules continue to emerge, holding promise for improved differentiation between demyelinating syndromes, refined outcome prediction, and enhanced therapeutic precision. Particularly promising, given their close ties to disease pathophysiology, are the assessment of cytokine and complement levels in both serum and CSF. In particular, the CSF concentration of IL-6 is increased in both NMOSD and MOGAD, distinguishing these two conditions from MS [96]. In contrast, serum levels of C3 and C4 are reduced during AQP4+NMOSD attacks but not MOGAD([97,98]). One study observed that CSF levels of C3 and C5 are increased in both conditions, while C5b-9 concentration was on average higher in NMOSD than MOGAD, and it was increased only in a minority of subjects with MOGAD and severe phenotype [99]. These findings offer a potential explanation for the discrepancies in complement deposition observed in pathology studies and suggest a possible role for complement activation in a subgroup of MOGAD patients with severe disease [100,101]. Although evidence supporting the use of these molecules as diagnostic and prognostic markers remains limited and at times conflicting, efforts to harmonize assay techniques, standardize timing, and account for treatment exposure at testing are essential for better result interpretation and the potential integration of these biomarkers into clinical practice.

## Other neuroinflammatory conditions

The pursuit to characterize autoimmune conditions with potentially causal antibodies has become a dominant theme in neuroimmunology. The attractiveness of a discrete answer as to the cause of a patient's neurological dysfunction is undeniable as treating patients with evermore potent immunosuppression warrants clear justification. Soluble biomarkers have been useful in understanding the pathophysiology, developing diagnostic criteria, and risk stratifying patients with other neuroinflammatory conditions aside from MS and NMOSD/MOGAD as well.

### Biomarkers in encephalitis

Encephalitis broadly refers to conditions that lead to inflammation of the brain. There may be a known etiology, for example a viral infection such as in HSV encephalitis, related to drug reactions such as with immune checkpoint inhibitors, or with confirmed or presumed autoimmune pathologies. In terms of soluble biomarkers, some conditions have a more specific antibody signature such as anti-N-methyl-d-aspartate (NMDA) receptor antibodies in NMDA receptor encephalitis (NMDARE), or anti-ganglioside GQ1b antibodies in Bickerstaff's encephalitis. Others may be elucidated by immunofluorescent patterns on rat or primate neural tissues that one day may be specified by techniques like antibody phage display, as was the case for Kelch-Like-11 rhombencephalitis [102]. Other autoimmune conditions still will have only associated antibodies, for example anti-Thyroid Peroxidase Antibodies in steroid-responsive encephalopathy associated with autoimmune thyroiditis, or no detectable antibodies at all, leaving gaps in defining diagnosis with an antibody-centric approach. Tracking response to immunotherapy and assessing relapse potential is also a challenge as patients with known causal antibodies like NMDARE will often have persistently detectable antibodies in serum and CSF following their recovery from an initial attack. However, in patients with NMDARE, the titre levels have been associated with a worse outcome as measured by the modified Rankin Scale (mRS) [103], yet as a measure of treatment response due to persistent of antibody despite treatment (even if effective), further study is needed [104]. Resultantly, research has diversified to explore alternative immunological factors.

A marker of neuroaxonal damage, which is a critical mode of damage in autoimmune encephalitis, is a rational tool to determine extent of damage in these conditions. Indeed, despite these conditions being very rare, multiple cohorts have illustrated that sNfL levels are elevated in patients with encephalitis, that higher levels may correlate with worse outcomes, and importantly that levels can remain elevated even when MRI imaging does not show significant worsening [87,105].

Cytokines and chemokines represent a promising group of biomarkers particularly where personalized tailoring of immunotherapy is concerned. IL1 (T cell co-stimulation, B-cell maturation/proliferation and cytokine release), IL6 (promotes plasma cell production and secretion), IL10 (Th1 to Th2 shift and cytokine production/regulation) and IL17 (a promoter of proinflammatory cytokine release) are among those most prominently implicated in neuroinflammatory disease. In some cases, these proteins can indicate disease activity and in others they may indicate prognosis. In MS, for example, one might be able to track a shift from Th1 associated cytokines interferon-gamma and tumor necrosis factor-alpha toward a Th2 dominated state with interleukins 4, 5, and 10 to indicate disease control or benign disease with the reverse heralding the type of disease activity that is more typically monitored for (i.e., new lesions on MRI, new symptoms, etc.) but its presence indicates a damaging failure of therapy has occurred [106].

### Biomarkers in neurosarcoidosis

Sarcoidosis is another incompletely understood inflammatory disorder where noncaseating granuloma deposition in tissues leads to recurring damage and flares. Sarcoidosis can affect the peripheral and central nervous system in 5–10 ​% of cases [107], and is found at autopsy in as high as 25 ​% of patients who may have had occult neurological involvement [108].

CSF studies have shown that elevated protein with moderate pleocytosis as well as hypoglycorrhachia are hallmark features, but that also oligoclonal banding and elevated IgG index may be present in 20–40 ​% of cases [108]. An elevated CD4:CD8 ratio of 3.5–5:1 or greater in the CSF non-specifically reflects enhanced activity of the adaptive immune system typical of autoimmunity although this ratio tends to more pronounced in granulomatous disease such as sarcoidosis rather than MS where the ratio is reversed [109,110]. This finding could in turn be linked to higher levels of CSF IL-6/IL-10 seen in neurosarcoidosis [111]. Another proposed pathophysiologically relevant biomarker was CSF angiotensin converting enzyme, however this has been reportedly not specific or sensitive in neurosarcoidosis [112]. In small cohorts, higher CSF NfL levels were helpful in distinguishing neurosarcoidosis from patients with extra-neurologic sarcoidosis, which logically is reasonable given peripheral and central nervous system injury can be seen in this manifestation of the disease [113].

Collateral serum biomarkers have been explored in autoimmunity with varying success. Serum Angiotensin Converting enzyme while often thought of as marker of granulomatous disease, has proven to be of little clinical value in diagnosing conditions like sarcoidosis. Soluble serum IL2 receptor levels on the other hand have shown some promise with acceptable performance characteristics in some series and a sensitivity of 88 ​% and specificity as high as 85 ​% [114].

In terms of cytokines, IL10 may be relatively elevated in patients anticipated to recover well from sarcoid granulomas [115]. Additionally, antibodies to or analogues of these cytokines have been proposed for various autoimmune conditions and response to therapy can be predicted in some cases by cytokine levels (e.g., IL6 receptor blockade superiority to adalimumab or MTX in rheumatoid arthritis when serum IL6 is elevated) [116,117]. Related cellular biomarkers have also shown promise where, for example, the monitoring for CD19/20 rebound following rituximab treatment can track with relapse risk in Aquaporin-4 mediated NMOSD (but notably not in MOGAD) potentiating reduced dosing and avoiding excessive immunosuppression in the former [118,119].

Despite advances in biomarkers giving indirect insight into the underlying pathology of the autoimmune condition, the standard of biopsy confirmation of certain diseases like granuloma for probable or definite neurosarcoidosis or in giant cell arteritis cannot yet be replaced. While only a minority of neuroinflammatory conditions lend themselves to direct cellular sampling of the inflammatory process through biopsy, the act of biopsy even still may expose patients to a high risk particularly where the pathology nears eloquent cortex or delicate anatomy such as brainstem or spinal cord. In such cases, looking for suitable alternative/systemic tissue evidence of inflammation could rely on CT of the Chest/Abdomen/Pelvis or gallium scan. Of note, while Positron Emission Tomography can provide increased resolution and sensitivity of 97 ​% for processes like systemic sarcoid greater than alternative imaging modalities such as gallium can, where the sensitivity is reported to be 88 ​%, limitations with respect to biopsy of sub-centimeter lesions may limit the usefulness of the test [120]. PET has also been used to demonstrate metabolic brain activity disturbance in encephalitis [121]. Both modalities can be used to track inflammatory disease activity although their non-specific and relatively expensive nature may limit them to adjunctive tests at best [122].

### Neuropsychiatric involvement in connective tissue diseases

Although several connective tissue diseases have reportedly involvement of the central nervous system, two with evidence of the utility of soluble biomarkers include systemic lupus erythematosus (SLE) and Sjogren's. There is limited evidence of how these conditions may impact the CNS, however sNfL has been used in both conditions to show that nervous system involvement is occurring [123].

In SLE multiple organ systems are affected by the development of multiple autoantibodies including double stranded DNA antibodies which result in altered clearance of nucleic acids, or anti-Smith antibodies, as well as elevated anti-nuclear antibodies which are a hallmark biomarker in the diagnostic criteria, in addition to reduction of complement proteins C3 and C4 and generation of anti-phospholipid antibodies (APLAS) [124]. The neurological manifestations of lupus can be complicated and mimic MS; as well, NMOSD and APLAS antibodies can co-exist further complicating this condition, and result in a picture similar to autoimmune encephalitis with psychiatric manifestations and seizures [[125], [126], [127]]. Several cohort studies have identified that sNfL levels increase in patients with neuropsychiatric involvement of SLE, and as this biomarker becomes more widely available it may be critical in assisting further investigations and potentially treatment decisions in patients with SLE [128,129].

Similar findings have been described in patients with primary Sjogren's syndrome, another multisystem connective tissue disease where lymphocytic infiltration of exocrine glands manifests with classic sicca symptoms of dry mouth, dry eye, and dry skin. Biological determination of this condition is based on elevated anti-Ro/SSA antibodies, though multiple other autoantibodies can be elevated in this condition as well, thus often specific gland imaging and biopsy are needed to confirm diagnosis [130,131]. The neurological manifestations of Sjogren's syndrome also includes peripheral nervous system involvement, including small fiber neuropathy and sensory ganglionopathy, as well as central nervous system involvements where patients can develop focal areas of demyelination in the brain or spinal cord mimicking multiple sclerosis, in addition to meningoencephalitis syndromes [132,133]. The incidence of neurological involvement in Sjogren's syndrome is estimated to occur in around 20 ​% of patients, and though there is limited data on biomarkers for neurological involvement small studies have again shown that elevated sNfL, which can reflect both central nervous system and peripheral nervous system damage, can be elevated may be useful as a screening tool [123]. Typically, if there is involvement of the brain or vascular system, which can lead to vasculitis and damage to peripheral nerves or brain tissue, treatment of these connective tissues diseases is escalated to cyclophosphamide containing regimens and involvement of neurology for frequent examinations and further testing may be indicated. Therefore, a role for sNfL as a soluble biomarker in multi-system diseases may be as a screening test for when to look further for neurological involvement.

### Neuro-Behcet's disease

Separate from connective tissues disorders includes vasculitides where inflammation involves blood vessel lining, walls, or perivascular areas and may involve vessels of the brain. Neuro-Behçet's disease (NBD) is an immune-mediated neurological disorder leading to headaches, cognitive or psychological disturbances, and focal neurological symptoms as a result of stroke. The underlying pathophysiology involves an aberrant immune response, where T-cell-mediated inflammation and cytokine dysregulation—particularly involving pro-inflammatory mediators like TNF-α and IL-6—play central roles. These immune processes can lead to inflammation of small- and medium-sized blood vessels within the CNS, causing endothelial injury that can lead to cerebral venous thrombosis, ischemia, and direct parenchymal damage. Though these are non-specific, these plus additional factors such as positive Pathergy testing, HLA-B51 presence, and the proper clinic syndrome can lead to a diagnosis of NBD [134]. The vasculitic mechanism is crucial for understanding the clinical variability and therapeutic challenges in managing the disorder.

Biomarkers are emerging as valuable tools in the diagnosis, prognosis, and assessment of treatment response in NBD, and are similar to those used in other neuro-inflammatory conditions. Elevated levels of cytokines—such as IL-6, IL-8, and TNF-α—in both cerebrospinal fluid and serum correlate with disease activity and severity, thereby offering potential markers for early diagnosis in cases where the clinical and radiological picture is unclear. Higher levels of CSF NfL have been shown to correlate with recurrent attacks and overall worse disability from inflammatory damage from NBD [135]. Additionally, indicators of endothelial dysfunction, including soluble vascular cell adhesion molecule-1 (sVCAM-1) and pentraxin-3 (PTX3), may help differentiate NBD from other neuroinflammatory conditions and monitor therapeutic efficacy [136]. Identifying both specific biomarkers to help differentiate inflammatory conditions as well as a combination of specific and non-specific biomarkers of neuraxonal damage that may indicate a more severe disease and prompt the need for higher efficacy treatments earlier on could tailor more personalized treatment strategies in these rare and disabling conditions.

### Inflammatory cerebral amyloid angiopathy

While the previously described biomarkers generally indicate autoimmunity in progress, genetics may indicate a risk of abnormal immune system response and can blur the line between degenerative and neuroinflammatory conditions. Inflammatory cerebral amyloid angiopathy (CAA) is a situation where patients develop not only a build up of amyloid protein in cerebral vasculature but also are at risk for inflammation of the perivascular area with lymphocytic infiltrates in the case of Cerebral amyloid angiopathy-related inflammation (CAAri), or of the vessel wall with granulomatous infiltrates in amyloid-beta (Aβ)-related angiitis (ABRA) [137]. In these condition the patients’ clinical picture is often a combination of neurocognitive symptoms in addition to focal neuro9logical changes, as well as imaging findings that can also demonstrate T2 hyperintensities typical of inflammatory changes. Certain biomarkers have been studied to investigation if the prognosis of patients with CAA can be predicted, such as amyloid-40, amyloid-42, total tau and NfL, and found that higher NfL confers a 2.15 increased hazard ratio of intracerebral hemorrhage recurrence [138]. Additionally, these more specific biomarkers of amyloid β have been investigated in this condition showing that amyloid-40 levels in the CSF may be negatively correlated with amyloid load measured via PET scans, and that in patients with inflammatory CAA there appears to be lower levels of CSF amyloid-42 than in patients with CAA without inflammatory changes [137]. Furthermore, patients may have genetic predisposition to developing these syndromes and have adverse reaction to treatments, such as APOE e4/e4 carriers who are at high risk for late onset Alzheimer disease as well as for autoimmune illness excessive reaction to anti-amyloid therapies (i.e., Amyloid Related Imaging Abnormalities – Edema (ARIA-E) or (ABRA)) [139]. On the surface one might suspect such carriers suffer from autoimmune vasculitis as a result of increase antigen presentation (i.e., via presentation of the excessive buildup of amyloid) but it is also apparent that they are more prone to immune dysregulation [140]. Of the potential genetic risk states for autoimmunity, major histocompatibility complex (MHC) haplotypes have been among the most studied. Although the specific haplotypes for inflammatory CAA have not been identified, in other conditions such as MS, for example, haplotypes HLA-DRB1∗1501, HLA-DR2, HLA-DR3 and HLA-DR4 associated are associated with increased risk of disease onset [141]. The sharing of these haplotype susceptibilities with other conditions like systemic lupus erythematosus supports that an idiosyncrasy with antigenic stimulation of the immune system may lead to broader tendency toward autoimmunity, and further study is needed to establish if the link between genetic predisposition and immune-mediated diseases can be predicted and intervened upon.

## Future of soluble biomarkers

Soluble biomarkers are revolutionizing the management of MS. Over the past eight years, advancements in highly sensitive analytical platforms, such as SIMOA, have enabled the reliable quantification of protein biomarkers in blood samples. sNfL exemplifies this progress, achieving FDA breakthrough device status on multiple platforms (Roche, Quanterix, Siemens Healthineers). sNfL has been implemented as an outcome measure in clinical trials, leading to regulatory approvals [142], and is increasingly adopted in routine clinical care. While soluble biomarkers have the potential to be vital in precision medicine for MS, their widespread implementation requires addressing technical, systemic, and patient-centric challenges.

**Fig. 2:**
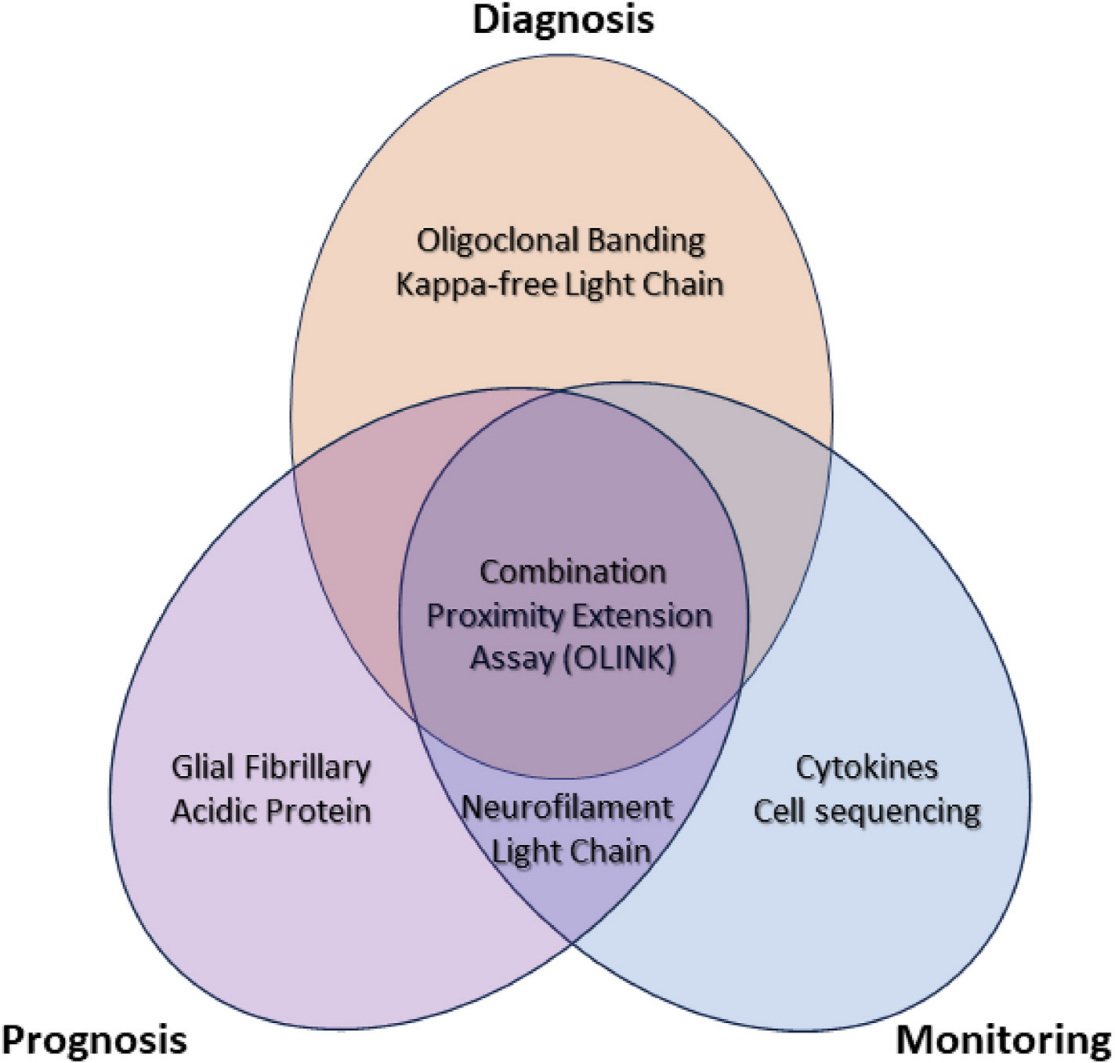
Clinical roles of soluble biomarkers in treating multiple sclerosis.

The integration of multiple biomarkers is pivotal for capturing the heterogeneity of MS, encompassing both inflammatory and non-inflammatory injury. Combining NfL (a proxy for relapsing pathophysiology) with GFAP (a marker of non-relapsing, progressive biology) has demonstrated prognostic superiority compared to either marker alone [30,43,60]. Moreover, CD40 ligand, a key mediator of T-B cell interactions, holds a dual role as a blood and CSF biomarker of lymphocyte activation linked to MS disease activity [143] and as a promising therapeutic target [144]. Beyond these, next-generation biomarker candidates include markers of lymphocyte function (e.g., CXCL13, sCD27), cytokines (e.g., IL-6, CXCL13), neuronal injury (e.g., NfH), microglial activity (e.g., sTREM2), and astrocyte activation (e.g., YKL-40) [42]. One notable commercially available multivariate blood biomarker tool that integrates markers of immune dysfunction and CNS tissue injury is the Octave(R) MS Disease Activity test. This composite of 18 proteins measured by OLINK protein extension assay, uses a proprietary algorithm and claims to be able to identify 4 discreate pathway signatures (immunomodulation, neuroinflammation, myelin biology and neuroaxonal injury) has shown promise [145,146]. Yet to be extensively externally validated, the company claims this test supports rational DMT treatment selection and inform disease activity monitoring. High-throughput “omics” technologies coupled with artificial intelligence are expected to identify optimal biomarker combinations (Fig. 2). Going one step further, integration of fluid biomarkers with multimodal approaches, such as clinical, imaging, electrophysiological, digital, and genetic data, promises comprehensive, patient-centered care tailored to the complexity of an individual's disease.

Despite their transformative potential, the adoption of soluble biomarkers faces significant hurdles. Informed by lessons learned with sNfL, technical challenges include adjusting for physiologic covariates, standardizing normative ranges across centers, addressing assay sensitivity variability, and ensuring platform interoperability. Rigorous validation across diverse populations remains a pressing need. Systemic barriers further constrain integration into practice, including high assay costs, lengthy regulatory processes, and a lack of widespread clinician training. From a patient perspective, convenience is critical—blood tests are preferred over invasive CSF sampling. Moreover, innovations such as point of care and even remote biomarker monitoring hold promise for revolutionizing long-term management, reducing hospital visits, and enabling continuous disease assessment.

Soluble biomarkers, led by sNfL and GFAP, are charting a path toward precision medicine and improved patient outcomes in neuroinflammatory diseases, and may have their greatest potential when used in combination for specific purposes. Their integration into clinical practice, alongside technological advancements and a patient-centered focus, is set to reshape management. Addressing current challenges through collaborative efforts in research, infrastructure, and policy will ensure their potential is fully realized.

## Author contributions

Gauruv Bose: Conceptualization, Methodology, Validation, Resources, Data Curation, Writing - Original Draft, Writing - Review & Editing, Visualization, Supervision.

Simon D.X. Thebault: Conceptualization, Methodology, Validation, Resources, Data Curation, Writing - Original Draft, Writing - Review & Editing, Visualization.

Giulia Fadda: Conceptualization, Validation, Writing - Original Draft, Writing - Review & Editing, Visualization.

John A. Brooks: Conceptualization, Validation, Writing - Original Draft, Writing - Review & Editing, Visualization.

Mark S. Freedman: Conceptualization, Methodology, Validation, Resources, Writing - Review & Editing, Visualization, Supervision.

## Declaration of competing interest

The authors declare the following financial interests/personal relationships which may be considered as potential competing interests: Gauruv Bose reports a relationship with Novartis Pharmaceuticals that includes: consulting or advisory and speaking and lecture fees. Simon Thebault reports a relationship with Novartis Pharmaceuticals that includes: consulting or advisory and speaking and lecture fees. Giulia Fadda reports a relationship with Novartis Pharmaceuticals that includes: consulting or advisory and speaking and lecture fees. Mark S. Freedman reports a relationship with Novartis Pharmaceuticals that includes: consulting or advisory and speaking and lecture fees. Gauruv Bose reports a relationship with EMD Serono Canada Inc that includes: consulting or advisory, speaking and lecture fees, and travel reimbursement. Gauruv Bose reports a relationship with Sanofi Genzyme Canada that includes: consulting or advisory, speaking and lecture fees, and travel reimbursement. Gauruv Bose reports a relationship with TEVA Pharmaceuticals that includes: speaking and lecture fees. Gauruv Bose reports a relationship with Multiple Sclerosis Society of Canada that includes: funding grants. Gauruv Bose reports a relationship with Innovation Fund that includes: funding grants. Gauruv Bose reports a relationship with Ontario Centre of Innovation that includes: funding grants. Simon Thebault reports a relationship with Sanofi Genzyme Canada that includes: consulting or advisory. Simon Thebault reports a relationship with Siemens Healthineers that includes: consulting or advisory. Simon Thebault reports a relationship with Innovation Fund that includes: funding grants. Simon Thebault reports a relationship with Multiple Sclerosis Society of Canada that includes: funding grants. Simon Thebault reports a relationship with American Brain Foundation that includes: funding grants. Giulia Fadda reports a relationship with Horizon Therapeutics Canada that includes: consulting or advisory. Giulia Fadda reports a relationship with American Brain Foundation that includes: funding grants. Giulia Fadda reports a relationship with Brain Canada Foundation that includes: funding grants. Mark S. Freedman reports a relationship with Sanofi Genzyme Canada that includes: board membership, consulting or advisory, funding grants, speaking and lecture fees, and travel reimbursement. Mark S. Freedman reports a relationship with Alexion that includes: board membership, consulting or advisory, and speaking and lecture fees. Mark S. Freedman reports a relationship with EMD Serono Canada Inc that includes: board membership, consulting or advisory, speaking and lecture fees, and travel reimbursement. Mark S. Freedman reports a relationship with Roche Pharmaceuticals - Hoffmann - La Roche Ltd that includes: board membership, consulting or advisory, and speaking and lecture fees. If there are other authors, they declare that they have no known competing financial interests or personal relationships that could have appeared to influence the work reported in this paper.
